# A systematic review of interventions to provide genetics education for primary care

**DOI:** 10.1186/s12875-016-0483-2

**Published:** 2016-07-22

**Authors:** Milena Paneque, Daniela Turchetti, Leigh Jackson, Peter Lunt, Elisa Houwink, Heather Skirton

**Affiliations:** i3S Instituto de Investigação e Inovação em Saúde, Universidade do Porto, Porto, Portugal; IBMC – Institute for Molecular and Cell Biology, UnIGENe and Centre for Predictive and Preventive Genetics (CGPP), Universidade do Porto, Porto, Portugal; Department of Medical and Surgical Sciences, Unit of Medical Genetics, University of Bologna, Bologna, Italy; Faculty of Health and Human Sciences, Plymouth University, Plymouth, PL4 8AA UK; Department of Clinical Genetics, Section Community Genetics, EMGO Institute for Health and Care Research, VU University Medical Center, Amsterdam, The Netherlands; Department of Family Medicine, School for Public Health and Primary Care, Maastricht University, Maastricht, The Netherlands

**Keywords:** Genetics, Professional education, Systematic review, Primary care

## Abstract

**Background:**

At least 10 % of patients seen in primary care are said to have a condition in which genetics has an influence. However, patients at risk of genetic disease may not be recognised, while those who seek advice may not be referred or managed appropriately. Primary care practitioners lack knowledge of genetics and genetic testing relevant for daily practice and feel inadequate to deliver genetic services. The aim of this systematic review was to evaluate genetics educational interventions in the context of primary care.

**Methods:**

Following the process for systematic reviews developed by the Centre for Reviews and Dissemination, we conducted a search of five relevant electronic databases. Primary research papers were eligible for inclusion if they included data on outcomes of interventions regarding genetics education for primary care practitioners. The results from each paper were coded and grouped under themes.

**Results:**

Eleven studies were included in the review. The five major themes identified inductively (post hoc) were: prior experience, changes in confidence, changes in knowledge, changes in practice, satisfaction and feedback. In five of the studies, knowledge of practitioners was improved following the educational programmes, but this tended to be in specific topic areas, while practitioner confidence improved in six studies. However, there was little apparent change to practice.

**Conclusions:**

There are insufficient studies of relevant quality to inform educational interventions in genetics for primary care practitioners. Educational initiatives should be assessed using changes in practice, as well as in confidence and knowledge, to determine if they are effective in causing significant changes in practice in genetic risk assessment and appropriate management of patients.

**Electronic supplementary material:**

The online version of this article (doi:10.1186/s12875-016-0483-2) contains supplementary material, which is available to authorized users.

## Background

It is estimated that approximately 10 % of United States (US) citizens [1] and 6 % of Europeans [2] are affected by a rare disease, equating to approximately 30 million individuals in the US [1] and the same number in Europe [2]. Of all rare diseases, 80 % have a genetic component [2] and it is therefore not surprising that at least 10 % of patients seen in the primary care setting are said to have a condition in which genetics has an influence [3]. However, there is evidence that patients at risk of genetic disease may not be recognised, while those who seek advice about their risks of rare genetic diseases may not be referred or managed appropriately by health professionals [4]. This may relate to lack of awareness of the family that a condition may have a genetic component, or may be due to lack of knowledge in primary care professionals that this may be so. A Dutch study in 2003 [5] showed that 70 % of patients believed that general practitioners (GPs) were not competent to handle queries about rare genetic conditions adequately, while in the US similar concerns about the competence of primary care practitioners to manage the patients with genetic concerns appropriately have been raised [6]. Undetected genetic risk can have serious consequences for the entire family, for example through preventing access to screening or preventive drugs or surgery (e.g. for cancer), resulting in increased morbidity, mortality, family burden and healthcare costs. Raising awareness of the risks and potential management of such cases is important for the patient and the wider family.

In the age of genomics, primary care practitioners will be increasingly involved in preventive care and organisation of relevant surveillance and develop a more flexible way to deal with patients’ requests for information and genetic tests. Primary care can be provided by a range of health professionals and is defined by the World Health Organisation as health care that is directly accessible by patients as the first point of contact, as well as being comprehensive and ongoing [7]. It involves prevention, and pre-symptomatic detection of disease, as well as early diagnosis [7], all of which are relevant to patients at risk of genetic disease. To illustrate this, Acheson et al. [8] reported that a sample of 100 primary care physicians had faced questions from patients related to familial cancer, neurological conditions, prenatal testing issues and a wide range of single gene disorders, confirming the need for knowledge and skill to deal with such queries. However, it has been reported that primary care physicians lack knowledge of genetics relevant for daily practice, lack oversight of genetic testing and feel inadequate to deliver genetic services [6, 9, 10].

As a result of a Delphi study involving experts in primary care education and genetic specialists in the United Kingdom (UK), Burke et al. [11] identified that primary care practitioners had three main responsibilities in relation to genetics: being able to identify patients at risk of a condition, contributing to medical management of such patients and appropriate communication of genetic information to patients. The authors of the paper developed a curriculum for genetics education in primary care organised according to these three activities [11]. However, genetics education is only very slowly starting to become a common part of medical curricula. This was made evident in a study by Julien-Reynier et al. [12], who studied the educational needs of 1170 GPs in five European countries and found that priorities for education differed according to country, gender and age of the physician. An average of 23.5 % (range 4.7–42 %) had received no genetics education in their undergraduate medical training, while another 29.7 % reported that the education they had received was not useful to them in their practice. A total of 984 (85.1 %) had not undertaken any continuing medical education related to genetics. Overall, the highest priority for education was ‘Genetics of Common Diseases’, followed by ‘Approaching Genetic Risk Assessment in Clinical Practice’, ‘Psychosocial and Counseling Issues’, ‘Basic Genetics and Congenital Malformations’, ‘Ethical, Legal and Public Health Issues’ and lastly ‘Techniques and Innovation in Genetics’.

However, provision of education may not always be sufficient to ensure improvements in patient care. In a paper on utilisation of research in practice, Grol and Grimshaw [13] point out that while many health professionals undertake continuing medical education, it is difficult to link this with changes in practice. This is borne out by Weinreich et al. [14], who instituted an educational programme focussed on improving detection of patients at risk of haemoglobinopathy, but found that while GPs regarded the education positively, this was not converted into changes in screening based on the patient’s ethnicity.

It is therefore clear that continuing medical education that is grounded in daily practice of GPs is urgently needed [9]. In order to provide effective genetics education, knowledge of the interventions that have been used in the past, and the outcomes of those interventions with their potential impact on health, is required. The aim of this study was to conduct a systematic review of educational interventions focussed on genetics and used in the context of primary care. The specific research question was: What are the outcomes of educational interventions in genetics for primary care professionals? As this was a mixed methods review, we used an inductive approach to search for possible outcomes. However, we anticipated that in line with the educational assessment framework proposed by Kirkpatrick [15] that is described later in this paper, outcomes would include satisfaction, changes in knowledge and skills and changes in behaviour.

## Methods

Conducting a systematic review enables the evidence on a particular topic to be gathered, analysed and synthesised. Adherence to a rigorous set of guidelines is essential to ensure rigour and objectivity. We followed the process for systematic reviews developed by the Centre for Reviews and Dissemination [16], which involved identification of relevant search terms, selection of studies based on explicit inclusion and exclusion criteria and quality assessment of papers (Table 1). The software package EndnoteX7 [17] was used to store, organise and retrieve all citations during the review process.

**Table 1:** Objective and Population, Intervention, Comparison, Outcomes, and Setting (PICOS) [18] elements

Review objective
To determine the outcomes of educational interventions in genetics for primary care professionals.
Participants
Sample of any size of primary care professionals who were recipients of an educational intervention focussed on genetics. Participants could be based in any country and be from any profession involved in delivering primary care
Intervention
Any educational intervention directed at qualified health professionals in primary care and focussed on genetic healthcare, including but not restricted to online or face to face education lasting one hour or more.
Comparators
None; control group of comparable professionals; control group of comparable practices.
Outcomes
Any outcomes, including but not restricted to: satisfaction with the educational intervention, changes in knowledge, changes in confidence changes in skills, changes in clinical behaviour, use of acquired genetics competencies, impact on organizational change or impact on patient health.
Study design
Any study design, including RCTs, quasi-experimental studies, cohort studies and qualitative studies.

### Search strategy

We conducted a search of five relevant electronic databases: Medline, CINAHL, SocIndex, PsychInfo and ERIC, using relevant search terms (see Table 2).

**Table 2:** Search terms

Genet^a^ OR genom* OR prenatal OR inherited
AND
Primary care OR general pract* OR family pract* OR community pract* OR midwi*
AND
Educat* OR train* OR teach*

The search terms were derived from numerous exploratory searches of the literature (as per CRD guidance) [16] and suggestions from experts in this field. We searched for the terms in titles and abstracts.

The search focussed on papers published between January 2005 and July 2015. The start date was chosen due to changes in the importance of primary care in genetic and genomic healthcare at that time, when the emergence of genomics in health shifted the focus of care in genetics from purely specialist services to primary and secondary care. For example, a seminal paper was published in 2003 urging the involvement of a wider health professional community in healthcare using genomics [19], while the same year there was recognition in the UK NHS White Paper [20] that primary care had a role in managing patients with genetic conditions, resulting in subsequent funding of posts for GPs with a special interest in genetics. It was therefore reasonable to expect that papers published in response to these initiatives might appear as early as 2005. We limited the search to studies that included human participants and were published in English. We also hand-searched the reference lists of relevant papers.

Papers were eligible for inclusion if they: i) were reports of primary research using qualitative, quantitative or mixed methods designs, ii) included data on outcomes (i.e. change in knowledge or change in practice) of interventions focussed on genetics education for primary care practitioners. Papers were excluded if they: i) focussed on health professionals other than those working in primary care, ii) focussed on patient education, iii) described an intervention but did not report outcomes, iv) reported educational needs without use of an intervention v) provided information on educational interventions but the results for primary care providers could not be separated from results for other professionals. For this purpose, we used the WHO definition of primary care and included practitioners from any healthcare profession who offered ongoing and comprehensive care at the first point of contact, directly accessible to patients [7].

As the purpose of a pilot study [21] is to assess the feasibility and logistics of a further (larger) research study, rather than to test an hypothesis and generate reliable and valid findings, we did not include pilot studies. Papers identified for possible inclusion were assessed by at least two researchers, who independently classified the papers and then discussed any differences of opinion until a consensus was reached [16]. A table of excluded papers and reasons for exclusion is available included as Additional file 1.

The eligible papers were assessed for methodological rigour by two researchers (HS and either DT or MP). Each researcher independently scored the paper using the system described by Kmet et al. [22]; scores were then compared and discussed until a consensus was reached. Kmet et al. [22] provide a checklist of 14 items for a quantitative study and 10 items for qualitative studies. A score of 2 (criterion fully met), 1 (criterion partially met) or 0 (criterion not met) is allocated to each item and the scores are totalled.

### Data extraction and synthesis

Data relating to the methods and main outcomes of each study were extracted and presented in a table. Relevant results or findings from each eligible study were coded and entered into a master table. The results/findings were grouped under themes that were identified inductively (post hoc) (see supplemental material for more detail on the codes and themes). Coding was undertaken by two researchers and reviewed by a third researcher. As the papers were heterogeneous in terms of methods, interventions and outcome measures, neither meta-analysis nor meta-synthesis was possible and we present the findings in a narrative form [16].

Because there were multiple outcomes for numerous studies, we reported every outcome and categorised these according to Kirkpatrick’s framework [15] for evaluating educational outcomes. This model can be used to distinguish the impact of education at four levels: valuation (level 1; satisfaction), learning (level 2; knowledge and knowledge retention), behaviour (level 3: applying knowledge and consultation skills regarding timely recognition of patients at risk) and effects on patient health and organization (level 4: changes in actual practice performance [i.e. referral] and results). The impact on society, or patient safety in genetic medical care, is part of level 4. The first learning step, according to Moore [23], would be to try and inform primary care health workers and aim for a better understanding of genetics. The first level of Kirkpatrick’s framework for evaluating the educational outcome then assesses satisfaction with the genetic modules. The higher the level, the more complex the potential learning outcome of the genetic module and the greater the possibility of having an impact on (genetic) health. We used Moore’s model of CPD curriculum design [23], identifying individual learning steps with their educational objectives and used the Kirkpatrick framework as a model to evaluate the genetic modules [15]. These analyses are presented in Table 5.

## Results

**Fig. 1:**
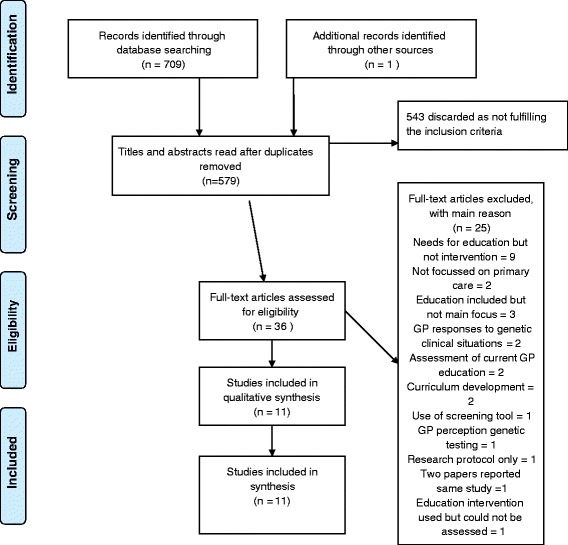
PRISMA 2009 flow diagram

There were twelve papers [24–35] identified for potential detailed review. However, two papers reported the same study [34, 35], so eleven papers were reviewed. The characteristics of the eleven individual studies [24–34] are presented in Table 3. The Preferred Reporting Items for Systematic Reviews and Meta-Analyses (PRISMA) flow chart [18] in Fig. 1 shows the selection process and the reasons for exclusion of papers. A list of excluded papers (with reasons for exclusion) is provided in the supplemental material. Regarding quality of the papers reviewed, the consensus quality score totals ranged (as a percentage of the maximum possible score) from 70 to 100 % and as we set the minimum acceptable score at 70 %, all eleven papers were deemed eligible for inclusion in the detailed review. We have included the scores and comments in Table 3 to enable judgements on the quality of the evidence to be made.

**Table 3:** Included studies

Authors, year, country, funding source (where available)	Aim	Methods and length of follow-up.	Participants	Intervention	Analysis	Main results	Scores (according to Kmet et al. [27] tool) and comments on quality issues
Bethea et al. (2008) [24] United Kingdom. Funding; UK Department of Health and NHS R&D.	To determine the current level of competence and confidence of primary care professionals in relation to management of familial cancers and explore how these were affected by educational outreach.	Quasi-experimental design. Longitudinal interventional study using matched groups. Baseline cross-sectional survey preceded the study. Follow-up data were collected six months after the completion of the intervention.	GPs and practice nurses from both rural and urban areas of England. 217 practitioners completed both pre and post intervention surveys: 29 from intervention group, 188 from non-intervention group.	Genetics educational outreach comprising two sessions on familial cancer and one on other genetic conditions (details reported in another paper [36])	Descriptive statistics to analyse data on confidence and competence. Logistic regression analysis to identify differences between ntervention and non-intervention groups.	Respondents from intervention practices more confident in risk assessment for breast cancer (OR 2.50, *p* = 0.03, 95 % CI 1.10–5.67) and in knowing family history to collect (OR 2.39, *p* = 0.02 95 % CI 1.14–5.00), make a risk assessment (OR 3.65, *p* = 0.01, 95 % CI 1.38–9.61)) and reassurance of low risk patients (OR 3.94,*p* = 0.01, 95 % CI 1.39–11.22) in relation to bowel cancer. Knowledge was improved in relation to correct assignation of breast cancer in the intervention group (*X* ^2^ = 4.37,*df* = 1, *p* = 0.04)	81 % Blinding of investigators not mentioned. Low response in pre- and post- intervention surveys from intervention group.
Carroll et al. (2009) [25] Canada. Funding; Ontario Womens’ Health Council.	‘To increase primary care providers’ awareness and knowledge of genetic issues and genetic services, as well as their confidence in dealing with genetic issues and use of resources.’	Quasi-experimental design. Longitudinal study using a survey pre-course (T1) and six months after the course (T3). Satisfaction with the programme was assessed immediately after the study day (T2).	Workshop attended by 29 primary care professionals but responses to survey from only 21(67 % were family physicians).	One day workshop for primary care professionals. Educational materials (powerpoint presentations) available after the workshop on the web.	Descriptive statistics. McNemar test and Wilcoxon signed rank test used to assess changes in knowledge and confidence between T1 and T3.	Self- assessed confidence in skills related to managing adult-onset conditions increased from pre-course mean score of 2.3 of a possible score of 5 (SD = 0.7) to post-course mean scores of 3.0 (SD = 0.9), (*p* = 0.005). Self- assessed confidence in skills related to prenatal genetics did not increase: the mean score pre-course was 3.4 (SD = 1.1) and post score was 3.6 (SD = 1.1). Knowledge increased regarding hereditary colorectal cancer: 5/21 attendees answered correctly pre-course, compared to 10/20 post course (*p* = 0.031).	72 % Research question and study design not well elucidated. Small sample no indication of required size.
Carroll et al. (2011) [26] Canada. Funding: Canadian Institutes of Health Research.	Evaluation of an educational intervention	Randomised controlled trial comparing family practitioners who received the intervention with those who did not. Pre-intervention data collected one month before intervention and post-data six months after intervention.	Family practitioners from a range of practices in Canada: 47 in intervention group and 33 in control group.	60 min workshop, portfolio of practical tools and knowledge support service	Analysis of covariance used to compare results in the two groups.	Those in intervention group scored more highly for confidence regarding referral decisions after the intervention. The adjusted mean score of the intervention group was 47.0 (95 % CI 44.9–49.2), compared with 37.9 (95 % CI 35.1–40.7) in the control group. They were more likely to make appropriate referral decisions with an adjusted mean of 7.8 (95 % CI = 7.4–8.2) in the intervention group, compared with mean of 6.4 (95 % CI = 5.8–6.9 in control group. The intervention group scored more highly on post-intervention knowledge questions, differences in knowledge scores between control and intervention groups indicated an odds ratio of 2.56 (95 % CI 0.90–7.31) for knowledge of the likelihood of a patient having a BRCA mutation, 1.43 (95 % CI 0.31–6.52) for percentage of women with breast cancer with a BRCA mutation and 1.23 (95 % CI 0.46–3.28) of number of patients with genetic predisposition to colorectal cancer who will develop the disease.	96 % Sample size small compared to that required to provide 80 % power to detect difference of 0.5 of a SD.
Clyman et al. (2007) [27] USA. Funding: US Department of Health and Human Services.	To assess the utility of an educational programme in medical genetics	Quasi-experimental design. Pre and post intervention survey. Knowledge tested immediately after each lecture.	36 GPs who had not had genetic education beyond basic medical training.	8 × 60 min lectures over 2 years and monthly 45 min seminars for two years.	Descriptive statistics and paired Student *t*-test.	There was an improvement in knowledge after the intervention, with mean pre-intervention score of 61.95 (SD 19.1, 95 % CI 58.8–65.1), compared with post-intervention scores of 76.1 % (SD 16.8, 95 % CI 73.3–78.9, 9), (*p* < 1×10^−10^).	70 % Sample small and analysis based on pre and post test score for each participant for each of 8 lectures to higher numbers.
Emery et al. (2007) [28] United Kingdom. Funding: Cancer Research UK and NHS R&D Support for Science.	Assessment of use of risk assessment software in conjunction with education.	Randomised controlled trial. Follow-up 12 months after intervention introduced.	45 practice teams – 23 in intervention group and 22 in control group.	45 min training session on cancer genetics. Lead clinicians for the research in each practice attended an additional 90 min session on using the software.	Use of software analysed using *t*-test. Linear mixed-effects models used to analyse referral appropriateness.	In intervention practices mean number of referrals was 6.2 (SD 3.1) per 10,000 registered patients per year, compared to a mean of 3.2 (SD 2.8) in control practices. The odds ratio of intervention vs control practices in referring patients in accordance with referral guidelines was 5.2 (95 % CI 1.7–15.8) and referred patients were more likely to have an increased risk of cancer when assessed by the genetic service (OR 0.7, 95 % CI 0.3–1.5	96 % Incomplete control of confounding variables.
Houwink et al. (2014) [30] Netherlands. Funding: Netherlands Genomics Initiative.	To determine whether primary care practitioners’ genetic knowledge improved longer term through an oncogenetics training programme.	A blinded, randomized controlled trial (RCT) comparing an intervention group (received education) and control group. Follow-up occurred six months after training.	80 Dutch GPs working in family practice: 40 in intervention group and 40 in control group. 24 from intervention group and 20 from control group completed the study	2 h online genetics education course.	Mean (test scores) and regression analysis.	More precise estimations of knowledge gained were obtained by the regression analysis. The effect of the intervention was found to be statistically significant, amounting to 0.055 on the proportion correct scale; the corresponding value for the standardized regression coefficient was .27, indicating an almost moderate effect size at T1. This value further increased 6 months after the intervention (.34, moderate effect size). Global score for satisfaction was 7.9/10 (SD 1.3, 95 % CI 7.5–8.3) while applicability scores were more diverse neutral scores for recognition of disease, referral to a specialist and knowledge of possibilities/limitations of genetic testing (2.7–2.9). The scores for increased knowledge about genetic diseases and basic genetic concepts were 3.3–3.8.	84 % Small sample size due to large attrition rate.
Houwink et al. (2014) [29] Netherlands. Funding: Netherlands Genomics Initiative.	To determine whether primary care practitioners’ genetic skills improved through an oncogenetics training programme.	A blinded, randomized controlled trial (RCT) comparing an intervention group (received education) and control group. Follow-up occurred three months after training.	56 (38 in intervention group, 18 in control group) GPs from two Dutch provinces.	4 h face to face training in oncogenetics.	Descriptive statistics and regression analysis.	Between group differences were found to be nonsignificant for the pretest (T0) and retention (T2) test, but the posttest (T1) difference of 0.19 in favor of the intervention group was found to be significant (*t*-test; *p* < 0.0005). Standardised regression coefficient for the effect of the training on skills was .34 at T1 (95 % CI .05–.23) one month after the training and .28 at T2 (95 % CI .03–.20) two months later, both indicating moderate effect size. Performance improvement in the intervention group at T2 was 80 % of the immediate effect at T1. Mean satisfaction and self-reported applicability scores were combined 7.7/10, SD 1.9, 95 % CI 6.7–8.6).	77 % High number of non-responders, high attrition rate, especially in control group.
Laberge et al. (2009) [31] US. Funding: Maternal and Child Health Bureau of the Health resources and Services Administration.	To evaluate the long term impact of the Genetics in Primary Care Project, a genetics educational programme to prepare primary care physicians for practice.	Qualitative descriptive study based on content analysis. Data collected during site visits or telephone to interview teachers. Follow-up period not exacty specified, but 3–4 years after completion of the programme.	Teams from 20 institutions.	Genetics in Primary Care Project programme included: 1) “train the trainer” workshops and (2) informal teaching in the medical school/residency curricula.	Content analysis.		75 % Little background connecting to theoretical framework or body of knowledge. Interviews described but content of site visits not clear.
Metcalfe et al. (2005) [32] Australia. Funding: no stated funding source.	To determine the effects of an educational intervention on GP knowledge of prenatal tests.	Quasi-experimental design. Single group pre-test and repeated post-test design. Questionnaire administered pre-workshop, post-workshop and 6–8 months later.	111 GPs who attended one of three workshops on prenatal testing. All provided antenatal care.	One face to face educational workshop based on two prenatal cases.	Frequencies and means. Student *t*-test to compare differences in means. Independent samples *t*-tests for comparisons between groups. ANOVA to identify factors influencing scores.	Number of GPs feeling quite or very confident about prenatal screening increased significantly (*p* < 0.01) from 15.9 % pre-workshop to 81.9 % post workshop and 45.1 % 6-8 months later. Knowledge of prenatal screening and testing was significantly improved from a mean of 51.2 % (SD 1.59, 95 % CI 48.02-54.39) pre intervention to 62.88 % (SD 1.51, 95 % CI 59.86-65.89) post workshop and 58.92 % (SD 1.6, 95 % CI 55.71–62.12) at follow up.	95 % Confidence was self-reported measure.
Srinivasan et al. (2011) [33] US. Funding: National Human Genome Research Institute.	To evaluate a web-based progamme on ELSI issues in genetics for primary care residents	Quasi-experimental design. Longitudinal study, using pre and post course surveys that covered prior experience, self-efficacy and knowledge of genetics. Follow-up data collected after completion of the curriculum (exact period of time not given).	210 primary care residents in paediatrics, internal medicine or family medicine, all were enrolled at one of three institutions.	Web-based educational programme based on ten cases and five tutorials. Each participant studied five cases and two tutorials.	Changes in self-efficacy and knowledge were analysed using the *t*-test. Anova was used to compare levels of experience in genetics between specialties. Descriptive statistics for non-parametric values.	Mean pre-test knowledge scores were 9.6/14, compared with post scores of 10/14. Overall self-efficacy increased from pre-course mean of 71.2 to post-course mean of 83.4.	73 % Little description of sample demographics. In two centres, the course was mandatory, which will have influenced uptake.
Wilson et al. (2006) [34] UK. Funding: NHS R7D Health Technology Assessment Programme.	To determine if GP confidence in managing patients with a family history of breast cancer was altered by use of educational session and a condition-specific software tool.	Pragmatic cluster randomised controlled trial. Intervention arm received software and training. Follow-up data were collected at the end of the one year intervention period.	GPs working in specific practices in a region in Scotland. Practices were assigned to either the control or intervention groups.	Software on CD-ROM was sent to each practice in intervention group. GPs in those groups were sent an individual letter about the study and invited to one educational session on use of software. Only 11.9 % of GPs in intervention group attended an educational session.	Chi-square tests, intracluster relation coefficient.	No significant differences reported between intervention and control groups in changes in GP confidence in managing patients, referral patterns, or initial patient risk assessment.	96 % Confidence in managing patients was self-reported measure.

### Overview of the studies

In the eleven papers included, three studies were undertaken in the United States (US) [27, 31, 33], two in Canada [25, 26], three in the United Kingdom (UK) [24, 28, 34], two in the Netherlands [29, 30] and one in Australia [32]. The style of delivery and topics covered are reported in Table 4. Six of the interventions were specifically aimed at GPs [26, 27, 29, 30, 33, 34]: the remaining four were aimed more generally at primary care professionals. In all studies, the intervention was assessed by testing changes in knowledge and/or practice. Houwink et al. [29] also tested the practitioner’s knowledge, attitudes and skills using a standardised patient. Laberge et al. [31] assessed the impact of their intervention on both clinical practice and genetic teaching of primary care specialists in university settings.

**Table 4:** Details of educational interventions by study

Study	Educational theory	Type of delivery	Focus	Content	Cases
Bethea et al. (2008) [24]	Educational outreach (Thomson et al. 2001 [36])	1. Tailored input into primary care practices by a genetic counsellor. 2. Face to face update sessions on relevant topics (3 sessions offered) 3. Practical tools such as referral guidelines and family history forms. 4. Opportunity for genetic counsellor to deal with patient queries.	To facilitate use of familial cancer guidelines and provide support to primary care on genetics issues.	Update sessions on: • Familial cancer referral guidelines • Risk assessment • Support and management of patients.	• Hereditary breast and ovarian cancer • hereditary bowel cancer cancer • haemochromatosis, cystic fibrosis, Marfan syndrome, fragile X and hypercholestrolaemia.
Carroll et al. (2009) [25]	Not stated	One-day face to face interactive workshop. Powerpoint files available online later.	To increase confidence of professional to become a resource on genetic health for other professionals in their communities	• Family history taking • Risk assessment • Referral to genetic services • Advantages and disadvantages of genetic testing • Obtaining resources • Ethical issues	• Alzheimer disease • hereditary breast cancer • hereditary colon cancer • cystic fibrosis • haemochromatosis • prenatal genetic screening.
Carroll et al. (2011) [26]	Not stated	One face to face interactive workshop session of 60 min. Portfolio of tools that could be used in primary care practice. Information sheet on current topics sent to participants every two weeks during the trial.	To improve referral decisions, confidence and knowledge relevant to primary care genetics.	• Practical medical genetics information • Risks, benefits and limitations of genetic testing including psychosocial risks, confidentiality and insurance issues.	• Hereditary breast and ovarian cancer • Hereditary colorectal cancer.
Clyman et al. (2007) [27]	Evidence based approach (Davis et al. 1995 [37])	Over two years, a 60 min face to face lecture each quarter (8 lectures in total) and a monthly 45 min didactic seminar.	To enable family physicians to provide appropriate services to patients with family or personal history of birth defects or mental retardation.	• Prenatal genetics • Maternal serum screening • Principles of medical genetics • Cytogenetics • Pedigree clinic • Dysmorphology in primary care • Genetics of common inherited disorders • Inherited cancer syndromes in primary care • Biochemical genetics • Genetic testing • Prenatal ultrasound anomalies	• States case-based approach but no examples given.
Emery et al. (2007) [28]	Not stated.	Computer decision support tool for familial cancer. One 45 min face to face educational session on familial cancer for medical and nursing staff in participating practices. One 90 min training session for lead clinician on use of the software.	Familial cancer risk assessment.	• Risk assessment for familial cancer • Referral guidelines for familial cancer.	None stated.
Houwink et al. (2014) [29]	Kirkpatrick (1967) [15] framework	4 h face to face training programme, including role play.	Oncogenetic consultation skills	• Family history taking • Genetic risk assessment • Referral • Ethical issues • Clinical knowledge required for cancer genetics.	Hereditary cancers: • Breast and ovarian cancer • Colorectal cancer • Skin cancer.
Houwink et al. (2014) [30]	Kirkpatrick (1967) [15] framework	2 h online course.	Oncogenetic consultation skills	• Family history taking • Genetic risk assessment • Common types of hereditary cancer • Referral guidelines • Possibilities and limitations of genetic testing • Clinical surveillance options	Hereditary cancers: • Colorectal cancer, • Breast and ovarian cancer.
Laberge et al. (2009) [31]	Not stated	Eight case-based modules delivered to trainers in a national workshop over a period of six months	Enhance faculty ability to teach genetics to primary care trainees.	Not supplied	Not supplied in the paper. Retrieved from Burke et al. (2002)[38, 42].
Metcalfe et al. (2005) [32]	Not stated	One case-based workshop (duration not stated). Participants also given an information pack including materials about the genetic service.	Enhance GP knowledge of prenatal tests in practice	• Prenatal counseling • Prevalence of birth defects • Counseling and informed choice • Screening for thalassaemia • Screening versus diagnostic testing • Pre-implantation genetic diagnosis	Two cases. Content not stated.
Srinivasan et al. (2014) [33]	Kern (1998) [38] - curricular development model	Interactive web-based programme.	ELSI genetics	• Core concepts in genetics • ELSI cultural issues • Medical ethics and law • Risk metrics/disease screening • Maternal fetal medicine.	• Alzheimer disease • androgen insensitivity • breast cancer • colon cancer • cystic fibrosis • Down syndrome • haemochromatosis • Huntington disease • Klinefelter syndrome • thrombophilia.
Wilson et al. (2005) [34]	Not stated	Educational session on cancer genetics and use of relevant clinical software.	Cancer genetics	• Referral guide • Genetic basis of cancers • Patient information • Contact information for local specialists.	• Breast and ovarian cancer • colorectal cancer.

The findings of the synthesis of evidence are presented under five main themes: prior experience, changes in confidence, changes in knowledge, changes in practice, satisfaction and feedback. A detailed table showing categories and themes derived from the paper is available in Additional file 2.

### Prior experience

Authors of only one study reported prior experience that could influence provision of genetic healthcare. Srinivasan et al. [33] found that only 69 (25 %) of the 279 residents training in primary care in their study had experience of genetic conditions amongst family and friends. Of those 69, 35 (12.5 % of total sample) had personal experience of involvement in the care of an affected person and over three quarters had cared for a patient with a genetic condition. While 55 % had ordered a genetic test, none had offered more than a few minutes of counselling to accompany those tests, although it is not stated what proportion of these tests may have been primarily to establish a diagnosis rather than being of a predictive nature. Of 111 GPs attending a workshop on prenatal tests [32], 40 % reported recording the full family history of the patient ‘always or often’ (p594).

### Changes in confidence in offering genetic healthcare

Practitioner confidence was shown to improve in six of the ten relevant studies, although the methods of assessing confidence differed across the studies. General practitioner confidence measured by Wilson et al. [34] did not significantly alter with respect to taking family history or ability to respond to patient queries about breast cancer risk. Similarly, only 23 % of the 22 practitioners interviewed by Laberge et al. [31] felt more confident in offering genetic healthcare.

However, Bethea et al. [24] reported participants in the intervention practices were more confident than controls (Odds Ratio (OR) 2.50, *p* = 0.03), especially related to family history collection (OR 2.39, *p* = 0.02), risk assessment (OR 3.65, *p* = 0.01) and reassuring those patients whom they assessed as low risk (OR 3.94, *p* = 0.01). While confidence scores increased across a range of 13 different topics when assessed by Carroll et al. [25], significant differences were found for only six activities or topics: assessing genetic risk (*p* = 0.033), making appropriate referrals (*p* = 0.033), discussing prenatal testing (*p* = 0.034), discussing benefits and limitations of genetic testing (*p* = 0.033), discussing content of a genetic counselling session (*p* = 0.022), and genetics of adult-onset disorders (*p* = 0.005) [25]. Participants in that study also gained confidence in being able to act as a resource for other colleagues with respect to genetics issues. Self-reported confidence in managing prenatal screening issues improved in the study by Metcalfe et al. [32], with 15.9 % of GPs feeling quite or very confident pre-workshop, compared with 81.9 % post-workshop and 45.1 % six to eight months later.

Those in the intervention group studied later by Carroll et al. [26] reported more confidence across a range of competencies (adjusted mean score of 37.9/55 in control group compared to 47.0/55 in intervention group) and scored higher than the control group on the topic of making referral decisions (7.8/10 compared with 6.4/10). Confidence was assessed in only the lead clinicians in Emery et al.’s study [28]: their self-reported confidence in managing patients with a family history of cancer improved after training (*p* < 0.001) and remained higher than baseline levels for the following twelve months.

Self-efficacy was measured in the study of medical residents by Srinivasan et al. [33], who reported that confidence in genetic skills increased from a mean of 71 (possible range 23–115) to a mean of 83.4. Specifically, mean scores for confidence in their own genetics knowledge was 11.7 pre-course (possible range of 4–20), rising to 14.1 post course, a significant difference (*p* < 0.01). However, as can be seen in the section below, this was not always matched by actual changes in knowledge as assessed by the researchers.

### Changes in knowledge

Authors of five studies reported improvements in knowledge of attendees. Carroll et al. [25] tested a small sample of 21 participants’ awareness of genetic services pre and post course and found no significant differences. Knowledge of breast and ovarian cancer was not shown to increase in participants, however a question on colorectal cancer was answered correctly by significantly more primary care providers (24 % pre-course increasing to 50 % post-course, *p* = 0.031). Although intervention participants had higher knowledge scores than controls in another study led by Carroll [26] (adjusted mean score of 47.0/55 in the intervention group compared to 37.9/55 in control group), the difference was not significant, nor was overall knowledge of genetics significantly changed in the study by Srinivasan et al. [33].

However, Houwink et al. [30] measured genetics knowledge using a test comprising 20 multiple choice items at three time points and found that knowledge had improved in the intervention group post-course and six months post-course. Professionals in participating practices correctly assigned risk in relation to familial breast cancer more frequently than those from non-participating practices (*p* = 0.04) in the study by Bethea et al. [24], while Clyman et al. [27] showed significant improvement in knowledge of family physicians post-course (*p* < 1×10^−10)^ and that they were less likely to over-refer or under-refer in the context of a hypothetical scenario. Metcalfe et al. [32] tested knowledge of prenatal screening, appropriate prenatal tests and risks of recessive inheritance and Down syndrome and the scores improved significantly from a mean of 51.2 pre-workshop to 62.88 post-workshop (*p* < 0.001) and 58.92 at follow up (*p* < 0.001).

### Changes in practice

Successful educational outcomes are those that lead to applications in practice. Such outcomes are difficult to assess when methods for reporting vary, and behaviour changes as a result of applying new knowledge may not reflect improvements in practice.

Houwink et al. [29] used standardised patient consultations (using trained actors) in a randomised controlled trial (RCT) to measure improvement in GP responses to familial cancer situations in practice pre-course, one month post-course and three months after oncogenetics training for GPs. Regression analyses revealed a moderate effect size for changes in performance in the intervention group between pre-test and post test score (standardised regression co-efficient = 0.34) and between one month post-test and three months post-test (standardised regression co-efficient = 0.28). Similarly, GPs who did a prenatal workshop were more likely to report discussing testing with all their pregnant patients (69.4 % pre workshop compared with 95.2 % post workshop and 85.7 % at follow up) [32].

Clyman et al. [27] showed no changes in referral pattern of course attendees. Wilson et al. [34] reported that patient referrals to genetic services did not significantly alter (*p* = 0.56) in the intervention group after the course and software package had been offered. However, those who had undergone the intervention were more likely to refer patients who were eventually assessed as having a high or moderate risk of inherited breast cancer, indicating a change in appropriateness of referrals. Referrals to cancer genetic services increased from those in the GRAIDS trial intervention practices [28], however when risk assessments of referred patients were made by specialist genetic staff, there were no differences found between risks to those referred by intervention practices and those referred by comparison practices.

When asked about whether they used the content of the programme in their practice following the course, 13 % of residents surveyed by Srinivasan et al. [33] replied that they used it often, 57 % occasionally and 29 % never. Houwink et al. [30] reported that 90 % of practitioners applied the knowledge at least once a month after the online course, 5 % indicated a frequency of use of at least once a week, while no participant reported daily application and another 5 % reported not encountering genetic issues in their practice. However, the authors did not assess the use of genetic knowledge prior to the course, so it is difficult to be certain how much difference the course actually made. In addition, while participants reported changes in knowledge, they did not report improvements in clinical skills. The primary care practitioners interviewed by Laberge et al. [31] were clinicians who were also involved in providing medical education. During telephone interviews with 22 respondents, 28 % reported changes in referral patterns for genetic healthcare, 23 % gave more consideration to genetics in the differential diagnosis, and 23 % said they put more emphasis on family history. Overall, 82 % indicated that the course had changed their clinical practice in some way, however this was not explored further. In another study^17^, 76 % (*n* = 32) attendees said their practice had changed a little, 21 % (*n* = 9) believed it had changed a lot. Where changes occurred, the nature of the change was unclear.

Primary care providers attending the course offered by Carroll et al. [25] were asked if their practice would change: 15 (86 %) intended to improve their family history taking and 10 (48 %) intended to increase their teaching activity in genetics.

### Satisfaction and feedback

Overall, participants reported satisfaction with the educational programmes studied. The residents in the study by Srinivasan et al. [33] reported that the programme delivered via the web offered more flexibility, but less contact with teaching staff than their usual programme. They did indicate that the web-based course was effective in teaching communication strategies, medical management and ethical, legal and social (ELSI) issues. The mean rating for satisfaction by GPs who took the course provided by Houwink et al. [29] was 7.7 of a possible 10, based on two Likert scales. In the other study by those authors [30], the mean satisfaction score was 7.9/10 (Standard deviation (SD) = 1.3). While participation in the genetics in primary care educational project [31] was seen as worthwhile by all participants, the additional workload and time required to participate were seen as reasons to advise colleagues to consider carefully before committing to it. The majority of those who evaluated the course offered by Carroll et al. [25] believed the course was relevant to them (24/28, 86 %) and that learning genetics with other primary care professionals was helpful (26/28, 96 %), while the Genetikit [26] course and materials were rated as either useful or very useful by over three-quarters of attendees. Eight lectures offered by Clyman et al. [27] were regarded as of good quality by 96 % of attendees and 81 % believed the course would be useful in practice and 97.1 % GPs attending the prenatal screening workshop provided by Metcalfe et al. [32] believed it would be useful for practice.

Finally, we present in Table 5 the findings of each paper aligned to the educational models of Kirkpatrick [15] and Moore [23], showing that authors of only four studies addressed Level IV, assessing organisational and healthcare impact.

**Table 5:** Analysis of papers using Kirkpatrick model

Kirkpatrick/Moore levels of education and evaluation	Kirkpatrick definition	Genetics module format	Assessment	Educational objective	Studies
I	Satisfaction	G-CPD or G-eCPD, live module, supportive website	Satisfaction, questionnaire	Information, understanding	Houwink et al. [29] Houwink et al [30]
II	Knowledge, self-reported competences of newly learned consultation skills	G-eCPD, G-CPD, live module	Multiple-choice questions, open ended questions, vignettes: pre/post and retention test	Information, understanding	Bethea et al. [24] Carroll et al. [25] Carroll et al. [26] Clyman et al. [27] Emery et al. [28] Houwink et al. [29] Houwink et al. [30], Laberge et al. [31] Metcalfe et al. [32] Srinivasan et al. [33] Wilson et al. [34]
III	Behavioural change	Live module	Responses to SP encounters in actual practice: pre/post and retention test	Synthesis, application, performance, attitude	Carroll et al. [26] Clyman et al. [27] Emery et al. [28] Houwink et al. [29] Laberge et al. [31] Srinivasan et a.l [33] Wilson et al. [34]
IV	Impact on organizational change and health gain, sustained change in practice behaviour and use of acquired genetics competencies	G-eCPD, live module, supportive website or other practical clinical genetic tool such as GenetiKit	GP referral data to genetics services	Analysis, synthesis, evaluation: health gain through timely (increased) referral to clinical genetics centers	Carroll et al. [26] Emery et al. [28] Laberge et al. [31] Wilson et al. [34]

## Discussion

The aim of this review was to ascertain the effectiveness of educational interventions on genetics for primary care. Practitioner confidence was positively affected in six of the eight studies where that factor was investigated, however even in those studies confidence was generally not enhanced across the entire range of possible topics. The interventions were therefore limited in increasing confidence. Knowledge of genetics topics improved in five studies, but again this tended to be subject specific and long term follow up indicated that the level of knowledge gain was not sustained. In only one of three RCTs where knowledge was tested were the changes significant. Overall, significant changes in practice were not demonstrated, with several studies showing no change and one RCT showing only a moderate effect size. While authors of some studies reported changes in practice, these were quasi-experimental or qualitative studies where the evidence was based on self-reports. While learners appeared satisfied with the programmes offered, the range of different questions posed by authors with respect to satisfaction makes the results difficult to interpret.

There are limitations to this study. Although the authors of the review followed a rigorous process to retrieve, select and analyse the available studies, the smallness of the samples and diversity of the studies made it difficult to draw coherent and robust conclusions from the evidence. In particular, we were unable to conduct a mathematical synthesis of the results. It should also be borne in mind that the search was restricted to papers published in English, so there may be evidence published in other languages that has not been included. We searched for peer-reviewed publications, so there may also be some bias due to the omission of unpublished data or results that were only available through the grey literature. However, the rigour of the study was enhanced by the use of more than one researcher to select, appraise and analyse the included papers and the search strategy was constructed based upon numerous trial database and hand searches, so we are reasonably confident that the relevant papers were retrieved.

Previous studies have cited that lack of knowledge about genetics issues, such as genetic tests, acted as a barrier to physician use [25]. In some studies included in this review, knowledge was not significantly altered: where changes occurred these tended to be as a result of prolonged exposure to genetics information, rather than short or one-off courses, but longer courses may be difficult for practitioners to access in terms of availability and time constraints. Some medical educators [39, 40] have suggested that ‘just in time’ information is required to ensure that practitioners can readily access information when their motivation is strongest, i.e. at the point of patient care. Patient queries appear to be related mainly to familial cancers, prenatal and reproductive questions and, less frequently, specific single gene disorders [25].

Raising primary care providers’ confidence through better organizational help and education would potentially enable them to apply genetics in practice, implement personalized genetic risk assessment for patients and potentially increase receptiveness to additional genetics education and training. However the self-confidence of primary care physicians in their ability to provide genetic healthcare is known to be generally low, for example Rinke et al. [41] found that although primary care paediatricians in the US reported that they frequently managed children with genetic conditions, they felt they lacked competence to deal with genetic issues. Many had however ordered genetic tests (although the authors did not distinguish between predictive and diagnostic tests) and referred patients to a genetic specialist: major reasons for referral were to obtain information about management and because of parental queries that they presumably felt they could not answer adequately. However, in this review we found that confidence of practitioners did generally improve following the educational programmes, in particular regarding genetic risk assessment. Improved confidence may well result in more appropriate referrals, rather than an increase in referrals, as practitioners become more certain of their ability to reassure patients at low risk. While many of the researchers in our review measured changes in referral numbers, further studies are needed to explore this in detail.

In the study by Burke et al. [11], risk assessment was seen by practitioners who were not genetic specialists as an important educational topic for them. Student motivation to learn is key to successful educational initiatives, and these results may indicate that primary care providers recognise risk assessment as a skill that is required to respond effectively to patients, and are therefore more willing to integrate the information on this topic into their practice. The fact that participants in one study [34] referred patients in the high risk group more appropriately for further genetic healthcare would seem to support this idea. Relevant to this point, Grol and Grimshaw [13] conclude that as well as educational opportunities, other strategies are also needed to support practical change, such as use of support at a policy level and provision of clinical decision aids. They also cite better reimbursement for genetics-related activities as an incentive, which concurs with the findings of Rinke et al. [41].

Three strong methodological issues emerge from this review. One is the difficulty in measuring the effectiveness of the learning activity. Some authors have measured changes in referral rate to genetic services, but in fact an increase in inappropriate referrals may indicate poor learning. Another strategy has been the observation of the proportion of trainees applying the learned knowledge [30], although, again, it is not necessarily a measure of the quality of their clinical application. A more nuanced approach than simply counting referrals is required. Wilson et al. [34], for example, investigated physician referrals based on how they classified their patients’ genetic risk(s) (low, moderate or high). Similarly, while confidence improved in most cases, changes in knowledge did not always alter significantly as a result of the educational intervention. This may be partly due to the difficulty in measuring changes in knowledge. Houwink et al. [29] tried to overcome this problem by using standardised patients to actually evaluate changes in practice, albeit with simulated patients. This approach would appear more valid than administration of a limited survey in which questions are often out of clinical context. Kirkpatrick’s model [15] for evaluation of educational interventions stresses that assessment of the impact of the learning on patient care is an important evaluative step, but this review indicates it is one that is rarely taken. This brings us to the last methodological issue clearly emerging from this review, which shows the highest educational outcome level (level IV) [15] (*organizational change and health gain, sustained change in practice behaviour and use of acquired genetics competencies*), is rarely assessed in studies evaluating impact of genetics education.

Although authors of four studies assessed outcomes at Kirkpatrick’s fourth level [15], they did not show clear evidence of improved impact on organizational change and health gain. One of the possible explanations could be that an increase in genetics knowledge and skills could also mean an increase in efficiency in referrals, not necessarily more or less referrals. Even if a scientific, logistical and ethical framework for the appropriate and effective use of genomic information in healthcare generally is in place, the primary care workforce is unlikely to be adequately prepared to provide such information in general practice. To do this, enhanced analysis using computer software may be helpful, but such systems must conform to regulations for preserving privacy of patients, and concerns about this aspect may be a barrier for integration of genetics and genomics in primary care [6]. As Rahimzadeh and Bartlett [6] also suggested, primary care providers may see genomics as conflicting with the holistic approach of primary care by reducing the patient to a unique molecular sequence. The same authors argue that the opportunities that genomic medicine presents for prevention and amelioration of disease fit well within the primary care framework and organisational change is needed to ensure that patients benefit from genomic advances.

If GPs stay genetically uneducated and therefore incompetent related to the use of genomic information in general practice, individual genetic medical care is likely to be unhelpful and may possibly be even harmful [13]. We believe the results of this review should be used in the near future to guide the implementation and level of assessment of genetics education internationally. Although the majority of the issues investigated cover genetics-related knowledge, skills and attitudes essential for every medical care provider, further studies will have to determine whether the results are relevant to other medical specialties as well.

## Conclusions

From this review it can be seen that long-term impact of genetics educational initiatives on patient management and policy has been poorly assessed and there is a dearth of high quality RCTs to provide a strong evidence base for educational practice. From the existing evidence, it appears that short-term educational initiatives alone are unlikely to cause significant changes in practice in the areas of recognition of genetic risk, assessment of risk and appropriate management of patients in patient care. This may in part be due to challenges in measuring change, but perhaps changes in knowledge should not be the main aim. Rather, effecting changes in genetic awareness and the ability to find the relevant information, when needed, may be better objectives. We therefore suggest that more research is undertaken to address whether, in addition to educational programmes aiming for high impact on organizational change and health gain, sustained change in practice behaviour and use of acquired genetics competences, provision of resources to supply ‘just in time’ information, decision aids and other clinical tools that are accessible during the clinical encounter contribute to changing primary care practice and genetic care improvement.

## Abbreviations

ELSI, ethical, legal and social issues; GP, general practitioner; OR, odds ratio; PICOS, patient population or problem, intervention (treatment/test), comparison (group or treatment), outcomes, and setting; PRISMA, preferred reporting items for systematic reviews and meta-analyses; RCT, randomised controlled trial; SD, standard deviation; UK, United Kingdom; US, United States.

## Additional files

Additional file 1: Papers read in full text but not included in the review. Table of papers excluded and the reason for exclusion. (PDF 97 kb) Additional file 2: Main themes and categories derived from the analysis. Table showing the main themes and all categories of data included in those themes. (PDF 26 kb)
