# Supplementary material for: A systematic review of interventions to provide genetics education for primary care
Source: BMC Fam Pract. 2016 Jul 22;17:89. doi: 10.1186/s12875-016-0483-2 (PMC4957387; doi:10.1186/s12875-016-0483-2)
Supplement: Additional file 1: — Papers read in full text but not included in the review. Table of papers excluded and the reason for exclusion. (PDF 97 kb) [file 12875_2016_483_MOESM1_ESM.pdf]

Additional material 1: papers read in full text but not included in the review

Reference	Reason for exclusion
Antoun et al (2010)[1]	Focussed on willingness for education
Barr et al (2007)[2]	Health visitor needs for education
Benjamin et al (2009)[3]	Midwife needs for education, not an intervention
Blaine et al (2008)[4]	Assessed appreciation of family history, not changes in knowledge or practice.
Blazer et al (2011)[5]	Mixed group of health professionals and relevant data for primary care could not be extracted.
Brannon et al (2011)[6]	Report of use of screening tool, not education
Calafato et al (2008)[7]	Reports needs for education, not intervention
Chen et al (2014)[8]	Focus on health educators, not professionals offering direct primary care
Drury et al (2007)[9]	Not reporting outcome of educational intervention (data reported in Bethea et al[10])
Dormandy et al (2012)[11]	Reporting mainly communication skills training in a genetic context.
Edelman et al (2014)[12]	Pilot study
Emery et al (2005)[13]	Protocol for study, results reported in Emery et al[14]
Feigenbaum et al (2014)[15]	Curriculum development
Flouris et al (2010)[16]	Questionnaire development
Fox et al (2008)[17]	Focus on service development
Gaff et al (2007)[18]	Not testing of an intervention
Harris et al (2006)[19]	Assessing educational needs.
Henriksson et al (2006)[20]	Not only focussed only on primary care and assessing content of current courses.
Houwink et al (2011)[21]	Needs for education, not an intervention
Houwink et al (2012)[22]	Needs for education, not an intervention
Julian-Reynier et al (2006)[23]	Not only focussed only on primary care and assessing content of current courses.
Julian-Reynier et al (2008)[24]	Focussed on educational priorities
Mainous et al (2013)[25]	GP perceptions of genetic testing
McCann et al (2005)[26]	Testing responses of GPs to genetic scenarios
Plass et al (2006)[27]	Assessing current education provision, not an intervention
Powell et al (2012)[28]	Focussed on education needs
Rafi et al (2013)[29]	Pilot study
Rinke et al (2014)[30]	Current practices in primary care
Trinidad et al (2008)[31]	Needs and attitudes, not educational intervention
Weinrich et al (2009)[32]	Not possible to ascertain effect of education on primary care team as paired responses for pre and post education not identifiable. Data included providers whom had education and those who didn't.
Westwood et al (2012)[33]	Impact of genetic services offered in primary care

Wilson et al (2006)[34]	Data reported in Wilson et al[35]

References

1. Antoun J, Zgheib NK, Ashkar K: Education may improve the underutilization of genetic services by Middle Eastern primary care practitioners. *Genetic Testing And Molecular Biomarkers* 2010; **14:** 447-454.

2. Barr O, McConkey R: Health visitors' perceived priority needs in relation to their genetics education. *Nurse Education Today* 2007; **27:** 293-302.

3. Benjamin CM, Anionwu EN, Kristoffersson U *et al*: Educational priorities and current involvement in genetic practice: a survey of midwives in the Netherlands, UK and Sweden. *Midwifery* 2009; **25:** 483-499.

4. Blaine SM, Carroll JC, Rideout AL *et al*: Interactive genetic counseling role-play: a novel educational strategy for family physicians. *Journal Of Genetic Counseling* 2008; **17:** 189-195.

5. Blazer KR, Macdonald DJ, Culver JO *et al*: Personalized cancer genetics training for personalized medicine: improving community-based healthcare through a genetically literate workforce. *Genetics In Medicine: Official Journal Of The American College Of Medical Genetics* 2011; **13:** 832-840.

6. Brannon Traxler L, Martin ML, Kerber AS *et al*: Implementing a screening tool for identifying patients at risk for hereditary breast and ovarian cancer: a statewide initiative. *Annals Of Surgical Oncology* 2014; **21:** 3342-3347.

7. Calefato J-M, Nippert I, Harris HJ *et al*: Assessing educational priorities in genetics for general practitioners and specialists in five countries: factor structure of the Genetic-Educational Priorities (Gen-EP) scale. *Genetics In Medicine: Official Journal Of The American College Of Medical Genetics* 2008; **10:** 99-106.

8. Chen L-S, Goodson P, Jung E *et al*: Effectiveness of a Web-based genomics training for health educators in Texas. *Genetics In Medicine: Official Journal Of The American College Of Medical Genetics* 2014; **16:** 271-278.

9. Drury N, Bethea J, Guilbert P, Qureshi N: Genetics support to primary care practitioners - a demonstration project. *Journal Of Genetic Counseling* 2007; **16:** 583-591.

10. Bethea J, Qureshi N, Drury N, Guilbert P: The impact of genetic outreach education and support to primary care on practitioner's confidence and competence in dealing with familial cancers. *Community Genetics* 2008; **11:** 289-294.

11. Dormandy E, Reid E, Tsianakas V, O'Neil B, Gill E, Marteau TM: Offering antenatal sickle cell and thalassaemia screening in primary care: a pre-post evaluation of a brief type of communication skills training. *Patient Education And Counseling* 2012; **89:** 129-133.

12. Edelman E, Lin B, Doksum T *et al*: Evaluation of a Novel Electronic Genetic Screening and Clinical Decision Support Tool in Prenatal Clinical Settings. *Maternal and Child Health Journal* 2014; **18:** 1233-1245.

13. Emery J: The GRAIDS Trial: the development and evaluation of computer decision support for cancer genetic risk assessment in primary care. *Annals Of Human Biology* 2005; **32:** 218-227.

14. Emery J, Morris H, Goodchild R *et al*: The GRAIDS Trial: a cluster randomised controlled trial of computer decision support for the management of familial cancer risk in primary care. *British Journal Of Cancer* 2007; **97:** 486-493.

15. Feigenbaum DF, Boscardin CK, Frieden IJ, Mathes EFD: What should primary care providers know about pediatric skin conditions? A modified Delphi technique for curriculum development. *Journal Of The American Academy Of Dermatology* 2014; **71:** 656-662.

16. Flouris A, Hawthorne G, Aitken M, Gaff C, Metcalfe SA: Development of a questionnaire for evaluating genetics education in general practice. *Journal Of Community Genetics* 2010; **1:** 175-183.

17. Fox R, Evans K, Bale S, Albury C, Tsepov D: Amniocentesis counselling: a role for the midwife-practitioner. *Journal Of Obstetrics And Gynaecology: The Journal Of The Institute Of Obstetrics And Gynaecology* 2008; **28:** 189-193.

18. Gaff CL, Aitken M, Flouris A, Metcalfe SA: A model for the development of genetics education programs for health professionals. *Genetics In Medicine: Official Journal Of The American College Of Medical Genetics* 2007; **9:** 451-457.

19. Harris R, Challen K, Benjamin C, Harris H: Genetic education for non-geneticist health professionals. *Community Genetics* 2006; **9:** 224-226.

20. Henriksson K, Kristoffersson U: Education in medical genetics for non-genetic health care providers in Sweden. *Community Genetics* 2006; **9:** 240-245.

21. Houwink EJ, van Luijk SJ, Henneman L, van der Vleuten C, Jan Dinant G, Cornel MC: Genetic educational needs and the role of genetics in primary care: a focus group study with multiple perspectives. *BMC Family Practice* 2011; **12:** 5-5.

22. Houwink EJF, Henneman L, Westerneng M *et al*: Prioritization of future genetics education for general practitioners: a Delphi study. *Genetics In Medicine: Official Journal Of The American College Of Medical Genetics* 2012; **14:** 323-329.

23. Julian-Reynier C, Arnaud S: France: genetics education for non-genetics health care providers. *Community Genetics* 2006; **9:** 227-234.

24. Julian-Reynier C, Nippert I, Calefato J-M *et al*: Genetics in clinical practice: general practitioners' educational priorities in European countries. *Genetics In Medicine: Official Journal Of The American College Of Medical Genetics* 2008; **10:** 107-113.

25. Mainous AG, 3rd, Johnson SP, Chirina S, Baker R: Academic family physicians' perception of genetic testing and integration into practice: a CERA study. *Family Medicine* 2013; **45:** 257-262.

26. McCann S, MacAuley D, Barnett Y: Genetic consultations in primary care: GPs' responses to three scenarios. *Scandinavian Journal Of Primary Health Care* 2005; **23:** 109-114.

27. Plass AMC, Baars MJH, Beemer FA, Ten Kate LP: Genetics education for non-genetic health care professionals in the Netherlands (2002). *Community Genetics* 2006; **9:** 246-250.

28. Powell KP, Christianson CA, Cogswell WA *et al*: Educational needs of primary care physicians regarding direct-to-consumer genetic testing. *Journal Of Genetic Counseling* 2012; **21:** 469-478.

29. Rafi I, Chowdhury S, Chan T, Jubber I, Tahir M, de Lusignan S: Improving the management of people with a family history of breast cancer in primary care: before and after study of audit-based education. *BMC Family Practice* 2013; **14:** 105-105.

30. Rinke ML, Mikat-Stevens N, Saul R, Driscoll A, Healy J, Tarini BA: Genetic services and attitudes in primary care pediatrics. *American Journal Of Medical Genetics Part A* 2014; **164A:** 449-455.

31. Trinidad SB, Fryer-Edwards K, Crest A, Kyler P, Lloyd-Puryear MA, Burke W: Educational needs in genetic medicine: primary care perspectives. *Community Genetics* 2008; **11:** 160-165.

32. Weinreich SS, de Lange-de Klerk ES, Rijmen F, Cornel MC, de Kinderen M, Plass AMC: Raising awareness of carrier testing for hereditary haemoglobinopathies in high-risk ethnic groups in the Netherlands: a pilot study among the general public and primary care providers. *BMC Public Health* 2009; **9:** 338-338.

33. Westwood G, Pickering R, Latter S *et al*: A primary care specialist genetics service: a cluster-randomised factorial trial. *The British Journal Of General Practice: The Journal Of The Royal College Of General Practitioners* 2012; **62:** e191-e197.

34. Wilson BJ, Torrance N, Mollison J *et al*: Cluster randomized trial of a multifaceted primary care decision-support intervention for inherited breast cancer risk. *Family Practice* 2006; **23:** 537-544.

35. Wilson BJ, Torrance N, Mollison J *et al*: Improving the referral process for familial breast cancer genetic counselling: findings of three randomised controlled trials of two interventions. *Health Technology Assessment (Winchester, England)* 2005; **9:** iii.
