# Supplementary material for: A systematic review of interventions to provide genetics education for primary care
Source: BMC Fam Pract. 2016 Jul 22;17:89. doi: 10.1186/s12875-016-0483-2 (PMC4957387; doi:10.1186/s12875-016-0483-2)
Supplement: Additional file 2: — Main themes and categories derived from the analysis. Table showing the main themes and all categories of data included in those themes. (PDF 26 kb) [file 12875_2016_483_MOESM2_ESM.pdf]

Additional material 2: Main themes and categories derived from the analysis.

Themes and categories
Prior experience<br>• Prior personal experiences of genetic condition<br>• Prior experience ordering a genetic test<br>• Prior experience caring for patients with genetic condition<br>• Prior experience referred patient for genetic counselling<br>• Prior knowledge of genetics professionals.<br>•
Changes in confidence<br>• Changes in confidence about genetic skills pre-education<br>• Changes in confidence in management of patients<br>• Changes in confidence ordering genetic tests<br>• Changes in confidence in providing counselling<br>• Changes in confidence to provide screening information<br>• Changes in confidence to provide psychosocial support after genetic test result<br>• Changes in confidence to be informal resource to other health professionals.
Changes in knowledge<br>• Changes in knowledge of genetics<br>• Changes in knowledge of genetic conditions<br>• Changes in understanding what happens in genetic services<br>• Changes in knowledge of prenatal issues<br>• Changes in awareness of need for counselling<br>• Changes in knowledge of genetic tests<br>• Changes in knowledge of genetic disease.
Changes in practice<br>• Application of knowledge<br>• Overall changes in clinical practice<br>• Changes in referral behaviour<br>• Changes in genetic risk assessment<br>• Changes in completeness of family history information<br>• Changes in contact with genetic services<br>• Changes in risk assessment of patients with cancer family history<br>• Changes in ability to discuss/refer to genetic services<br>• Increased awareness of genetics when seeing patients<br>• Changes in importance given to family history<br>• Changes in use of family history.

Satisfaction and feedback<br>• Satisfaction with course<br>• Views on web-based course delivery.
Other<br>• Use of software for family history cancer assessment<br>• Changes in patient knowledge<br>• Changes in patient risk perception<br>• Changes in attitude to genetic testing.
